# Current Knowledge on the Biology of Lysophosphatidylserine as an Emerging Bioactive Lipid

**DOI:** 10.1007/s12013-021-00988-9

**Published:** 2021-06-15

**Authors:** Jumpei Omi, Kuniyuki Kano, Junken Aoki

**Affiliations:** 1 Department of Health Chemistry, Graduate School of Pharmaceutical Sciences, The University of Tokyo, Tokyo, Japan; 2 AMED-LEAP, Japan Agency for Medical Research and Development, 1-7-1 Otemachi, Tokyo, Japan

**Keywords:** Lysophospholipid, Lysophosphatidylserine, GPCR, Phospholipase, Immune regulation

## Abstract

Lysophosphatidylserine (LysoPS) is an emerging lysophospholipid (LPL) mediator, which acts through G protein-coupled receptors, like lysophosphatidic acid (LPA) and sphingosine 1-phosphate (S1P). LysoPS is detected in various tissues and cells and thought to be produced mainly by the deacylation of phosphatidylserine. LysoPS has been known to stimulate degranulation of mast cells. Recently, four LysoPS-specific G protein-coupled receptors (GPCRs) were identified. These GPCRs belong to the P2Y family which covers receptors for nucleotides and LPLs and are predominantly expressed in immune cells such as lymphocytes and macrophages. Studies on knockout mice of these GPCRs have revealed that LysoPS has immune-modulatory functions. Up-regulation of a LysoPS-producing enzyme, PS-specific phospholipase A_1_, was frequently observed in situations where the immune system is activated including autoimmune diseases and organ transplantations. Therefore, modulation of LysoPS signaling appears to be a promising method for providing therapies for the treatment of immune diseases. In this review, we summarize the biology of LysoPS-producing enzymes and receptors, recent developments in LysoPS signal modulators, and prospects for future therapeutic applications.

## Introduction

**Fig. 1:**
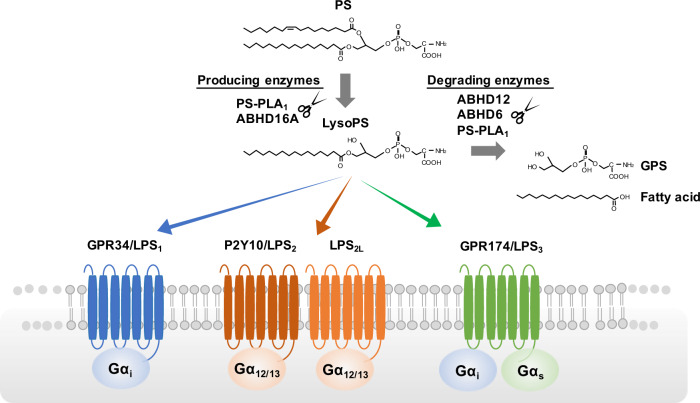
Receptors and metabolizing enzymes for LysoPS. LysoPS is enzymatically produced from PS by PLA reaction mediated by PS-PLA_1_ or ABHD16A. Produced LysoPS can activate four GPCRs, GPR34/LPS_1_, P2Y10/LPS_2_, LPS_2L_, and GPR174/LPS_3_. These LysoPS receptors are mainly expressed in the immune cells and exert a variety of immunological functions through the activation of downstream Gα proteins. LysoPS is subsequently degraded into glycerol-3-phosphoserine (GPS) and fatty acid by ABHD12, ABHD6, and PS-PLA_1_

Lysophospholipid (LPL) is a minor phospholipid with a single fatty acid at the *sn*-1 or *sn*-2 hydroxide of a glycerol backbone. Depending on their polar head and type of fatty acid, LPLs comprise a diverse range of molecules, with hundreds of different species. LPLs are produced in various physiological and pathological contexts and evoke a wide variety of cell responses by activating G-protein-coupled receptors (GPCRs) specific to each LPL type. Among them, lysophosphatidic acid (LPA) and sphingosine-1-phosphate (S1P) has been extensively characterized over the past two decades, and thus are now well-established as important bioactive lipids or lipid mediators in pathophysiology and also as drug targets [1, 2]. Another LPL that has recently attracted much attention is lysophosphatidylserine (LysoPS), which has phospho-L-serine as a head moiety. Little had been known about its role as a lipid mediator because it was a minor phospholipid in tissues and its receptors had been poorly understood. Recent great advances in mass-spectrometry techniques have overcome the former problem, and we now know that LysoPS is present in the central nervous system (CNS) and immune system [3]. Several groups including our group have identified specific receptors for LysoPS, i.e., GPR34, P2Y10, and GPR174. We have proposed that these receptors should be designated as LPS_1_, LPS_2_, and LPS_3_, respectively, according to the nomenclature of the LPA and S1P receptors. In this review, we use these terms for the three LysoPS receptors. Recent analyses of mutant mice of these LysoPS receptors revealed immunomodulatory functions of LysoPS (Fig. 1) [4–11]. Moreover, LysoPS-metabolizing enzymes, including producing enzymes and degrading enzymes, have been identified in platelets and the CNS, leading to a better understanding of the links between LysoPS and diseases such as neurodegenerative disorders and autoimmune diseases [12–19]. Herein, we provide a comprehensive review of the current knowledge on tissue distribution, mechanisms of action, production and degradation, and biology of LysoPS with its clinical implications.

## Structural Variety and Tissue Distribution of LysoPS

LysoPS is a deacylated form of phosphatidylserine (PS) and has a fatty acid at either the *sn*-1 or *sn*-2 position of the glycerol backbone. As in the case of other LPLs, LysoPS detected in vivo is composed of several LysoPS species with a fatty acid varying in carbon chain length and degree of unsaturation (e.g., C16:0 (palmitic acid), C18:1 (oleic acid), and C22:6 (docosahexaenoic acid), etc.). These LysoPS species can be detected and quantified easily using liquid chromatography (LC) linked to the latest mass spectrometry (MS) (LC-MS) with highly improved sensitivity [20]. Okudaira et al. [21] reported that non-enzymatic conversion of 2-acyl-1-LysoPS to 1-acyl-2-LysoPS, which is referred to as intramolecular acyl-migration, is completely inhibited in mild acidic condition (pH 4), enabling the accurate quantification of 1-acyl- and 2-acyl-LysoPS in biological samples. Indeed, they showed that both 1-acyl- and 2-acyl-LysoPS were present in various murine tissues at similar levels. Interestingly, acyl-species bias was detected: while the majority of 1-acyl-2-LysoPS have a shorter, saturated (or monounsaturated) fatty acid such as C16:0, C18:0 or C18:1, the majority of 2-acyl-1-LysoPS have a longer, poly-unsaturated fatty acid such as C18:2, C20:4 or C22:6. These established techniques are now available and indispensable to understand the biological significance of LPLs, including LysoPS, since the receptor-activating potency often differs among LPL species, even between the *sn*-1- and *sn*-2-acylated isomers [22].

Analyses using the above-mentioned LC-MS method revealed that various LysoPS species were detected in all the tissues and cells tested. LysoPS species are comprised of 18:0-, 18:1-, 18:2-, 20:4- and 22:6-LysoPS depending on the sources. The total amount of LysoPS species detected was 1–10 μg/g tissue in major organs, including brain, heart, lung, liver, spleen, and kidney. Of note, LysoPSs are relatively abundant in immune-related organs (spleen, thymus, and lymph node), CNS (brain and spinal codes), liver, and also colon. Interestingly, the LysoPS level was dramatically higher in activated immune cells. Shinjo et al. demonstrated that T cell stimulation by anti-CD3/CD28, which mimics the activation via a T-cell receptor (TCR), led to significant production of LysoPS species [4]. Similarly, lipopolysaccharide (LPS), a strong ligand for Toll-like receptor 4, also caused the accumulation of LysoPS species in thioglycolate-elicited peritoneal macrophages [13]. Interestingly, almost half of the produced LysoPS in LPS-stimulated macrophages was subsequently secreted to the culture supernatant whereas the LysoPS produced in activated T cells was exclusively retained in cells, suggesting that the mechanism for the LysoPS production is cell type-specific. Nevertheless, these findings raise the possibility that LysoPS functions in immune-related contexts including both innate and humoral immune responses.

LysoPS is also present in the blood in small amounts. Under normal conditions, plasma LysoPS levels are extremely low, on the order of a few nM in total [3, 21]. In contrast, serum LysoPS levels are high, suggesting that LysoPS is produced upon blood coagulation, in which activated platelets could be a source of PS.

## Synthetic and Degradative Pathways of LysoPS

Unlike LPA which can be synthesized de novo by acylation of glycerol 3-phosphate, LysoPS is produced by de-acylation of PS. Phospholipase A_1_ and A_2_ (PLA_1_/PLA_2_) catalyze the hydrolytic cleavage of ester linkages between the glycerol-3-phosphoserine backbone and the fatty acid at the *sn*-1 and *sn*-2 position, respectively. These reactions occur either intra- or extracellularly. Our group identified two PLAs, group IIA secreted PLA_2_ (sPLA_2_-IIA) and phosphatidylserine-specific PLA_1_ (PS-PLA_1_), as candidates of such extracellular PLAs [23, 24]. More recently Cravatt’s group identified α/β-hydrolase domain-containing protein 16 A (ABHD16A) as a candidate of an intracellular LysoPS producing enzyme, and ABHD12 and ABHD6 as LysoPS degrading enzymes [13, 19, 25]. Since the enzymatic characteristics, biological and pathological roles of these enzymes have been elegantly described in [26], we briefly mention their properties.

## Phosphatidylserine-specific PLA1 (PS-PLA1)

PS-PLA_1_, also known as PLA1A, is a member of the pancreatic lipase family, whose activity was first detected in the supernatant of activated rat platelets [23]. PS-PLA_1_ has a catalytic triad composed of Ser, Asp, and His, which is shared in the majority of serine hydrolases, three putative N-linked glycosylation sites, and a hydrophobic signal peptide at its N-terminal position, which is required for its secretion into extracellular spaces [27]. The most prominent feature of PS-PLA_1_ is that it is specific to PS and that, unlike other lipases, never hydrolyzes triacylglycerol (TAG) [23]. The loop structure surrounding the active site called a “lid”, is composed of a shorter chain of amino acids in PS-PLA_1_ than in other pancreatic lipases with TAG lipase activity. The short lid structure was also observed in phosphatidic acid (PA)-selective PLA_1_α (PA-PLA_1_α/LIPH), guineapig pancreatic-related lipase, and many insect PLA_1_s, all of which showed greater PLA_1_ activities than TG lipase activities. Thus, the short lid may somehow contribute to PLA_1_ molecules’ recognition of PLs rather than TGs.

Genetic studies on human congenital wooly hair (hairless disease) and analyses of mouse mutants revealed that the above-mentioned PA-PLA_1_α is one of the enzymes responsible for supplying a ligand, i.e., LPA, to its downstream LPA receptor, LPA_6_. PA-PLA_1_α is the enzyme that shows the highest homology to PS-PLA_1_. Therefore, PS-PLA_1_ was hypothesized to be a LysoPS-producing enzyme. This hypothesis was confirmed by the fact that a recombinant PS-PLA_1_ could produce LysoPS and activate LysoPS receptors when LysoPS receptor-expressing cells were treated with the enzyme. In addition, the recombinant PS-PLA_1_ protein could stimulate the degranulation of rat mast cells by producing LysoPS [28].

Min et al. suggested that PS-PLA_1_ is used by viruses for their assembly and replication. They showed that PS-PLA_1_ is significantly upregulated upon hepatitis C virus infection in a human hepatocellular carcinoma cell line and supports HCV assembly and replication through its interaction with viral membrane proteins NS2 and NS5A [29]. LysoPS itself partially rescues the knockdown effect of PS-PLA_1_, suggesting that LysoPS produced by PS-PLA_1_ somehow contributes to HCV assembly and replication. In this context, however, the responsible LysoPS receptors remain to be elucidated in Table 1. The same group also demonstrated the role of PS-PLA_1_ in anti-viral cellular responses [30]. Overexpression of PS-PLA_1_ enhances the anti-viral type-I interferon response, which is induced by the infectious Sendai virus, while PS-PLA_1_ knockdown reduces the response. Treating the cells with LysoPS, however, did not rescue the effect of PS-PLA_1_ knockdown. Thus, in this context, the anti-viral function might be independent of the enzymatic activity of PS-PLA_1_.

**Table 1:** Pathophysiological functions of LysoPS receptors

Receptor	Experiment	Model or cell-types	Functions	Refs.
GPR34/LPS_1_	In vivo	DTH model	Suppress pro-inflammatory cytokine production	[43]
	In vivo	Fungal infection model	Promote fungal clearance	[43]
	In vivo	Neuropathic pain model	Enhance microglial pro-inflammatory responses	[46]
	In vitro	Primary microglia	Promote phagocytosis	[45]
	In vitro	Cervical cancer cell gastric cancer cell colorectal cancer cell	Promote cellular invasion and proliferation	[47, 48]
P2Y10/LPS_2_	In vitro	Primary dendritic cell, microglia	Suppress cytokine production	[7]
	In vitro	Primary eosinophil	Promote degranulation, survival, and formation of EET	[5, 8]
GPR174/LPS_3_	In vivo	EAE model sepsis model	Attenuate disease severity by suppressing Treg cell function	[3]
	In vivo in vitro	Primary CD4 T cell	Suppress IL-2 production	[6]
	In vivo	Splenic follicular B cell	Inhibit the cellular migration into the follicle center	[9]
	In vivo	Splenic marginal zone B cell	Inhibit the inflammatory responses, proliferation, and differentiation	[11, 4]

**Table 2:** Current development of LysoPS analog

Compound	Target	EC50 (nM)				
		LPS1	LPS2	LPS3	Mast cell degranulation	Refs.
	Pan-agonist	230–550	20–28	300–520	150	[44, 70, 71, 75, 76]
	LPS1, LPS2	360	93	Inactive	n.t.	[70]
	LPS2, Mast cell	>1 μM	25–30	Inactive	200	[70, 71, 76]
	LPS1	37	>1 μM	Inactive	n.t.	[75]
	LPS1	5	>3 μM	Inactive	n.t.	[74]
	LPS2	Inactive	6.7	Inactive	n.t.	[75]
	LPS2	>1 μM	3.3	>1 μM	n.t.	[71]
	LPS3	Inactive	>1 μM	31	n.t.	[71]
	Mast cell	Inactive	>1 μM	>3 μM	10	[70, 76]
	Mast cell	Inactive	Inactive	inactive	3	[76]
	LPS2, Mast cell	>1 μM	1.7	>1 μM	300	[71, 76]

## ABHD16A and ABHD12

In the past decade, Cravatt’s group has reported several novel enzymes belonging to the α/β-hydrolase domain (ABHD) protein family as candidates for LysoPS metabolizing enzymes. Using pharmacological approaches, Juha et al. [14] first identified ABHD16A, also known as BAT5, as a serine hydrolase that is highly expressed in the brain. They showed that it preferentially catalyzed the hydrolysis of monoacylglycerol (MAG) with an unsaturated long-chain fatty acid, but not diacylglycerol (DAG) or TAG. Subsequently, Kamat et al. proposed that ABHD16A functions as a PLA rather than a lipase. Indeed, the greatest hydrolysis activity against PS was detected in the membrane fraction of cells overexpressing ABHD16A, although substantial hydrolysis activities were also detected for other phospholipids such as PC, PE, and PG [13]. Interestingly, in the brain of ABHD16A KO mice and in cells treated with shRNAs for ABHD16A, the level of PS, but not the levels of PC, PE, or MAG, increased with a concomitant decrease in the LysoPS level, suggesting that ABHD16A hydrolyzes PS to produce LysoPS in intact cells. It should be noted here that ABHD16A is an intracellular enzyme and produces LysoPS intracellularly.

Several groups have identified ABHD12 and ABHD6 as lysophospholipases that degrade LysoPS. These enzymes were initially characterized as hydrolyzing enzymes for 2-arachidonoyl glycerol (2-AG), a major endogenous ligand for cannabinoid receptors that negatively regulates endocannabinoid signaling in the CNS. *ABHD12* has been subsequently identified as a causal gene for the neurodegenerative disorder, polyneuropathy, hearing loss, ataxia, retinitis pigmentosa, and cataract (PHARC) [31]. Subsequently, Blankman et al. demonstrated that ABHD12-deficient mice develop PHARC-related phenotypes but show only minor changes in 2-AG metabolism in the brain, suggesting that other substrates may be responsible for PHARC development in vivo [19]. Based on an untargeted metabolomic analysis, the authors found that ABHD12-deficient mice accumulated significant amounts of LysoPS in the brain. Similarly, pharmacological inhibition of ABHD12 resulted in a significant accumulation of LysoPS in human macrophages and a significant enhancement of the immune responses of the cells in vivo [17]. Importantly, Kelkar et al. later showed that ABHD12 also catalyzed the hydrolysis of an oxidized-PS to LysoPS which was further hydrolyzed and degraded by ABHD12 [32]. More recently, LysoPS with a very-long-chain fatty acid (~C24:0), which is a preferential substrate for ABHD12, was shown to efficiently induce pro-inflammatory responses of macrophages via Toll-like receptor 2 (TLR2) [15]. These findings emphasize new aspects of PHARC as an immune-related disorder, and further support the immunopathological significance of LysoPS and its receptors, as described below. Like ABHD16A, both ABHD12, and ABHD6 are intracellular enzymes and thus act on LysoPS intracellularly. It is thus unclear how LysoPS produced and degraded by these intracellular enzymes is involved in extracellular signaling through GPCR-type LysoPS receptors.

## LysoPS Receptors

LysoPS was initially identified as a bioactive lipid that strongly enhances IgE-triggered histamine degranulation in rodent mast cells [33–35]. Several studies in the 1990s and 2000s suggested that LysoPS has multiple functions, including enhancing nerve growth factor-induced neurite outgrowth in PC12 cells [36], inhibiting the mitogen-induced human T cell proliferation [37], inducing the chemotactic migration of human glioma cells and murine fibroblasts [38], and activating TLR2 on dendritic cells (DCs) [39]. These studies implicated the presence of specific receptors for LysoPS. Several groups including our group have identified LysoPS receptors. These include GPR34/LPS_1_, P2Y10/LPS_2_, A630033H20Rik/LPS_2L_, and GPR174/LPS_3_, all of which are close homologs of the LPA receptors P2Y9/LPA_4_, GPR192/LPA_5_, and P2Y5/LPA_6_. Thus, both LPA and LysoPS are recognized by similar GPCRs belonging to the P2Y family. In the past decade, four LysoPS receptors have been functionally characterized as described below.

## GPR34/LPS_1_

LPS_1_ was initially found in a human brain cDNA library based on its sequence similarity with the platelet-activating factor (PAF) receptor [40, 41]. In 2006, Sugo et al. screened a large chemical library based on their inhibitory potencies against forskolin-induced cAMP accumulation in human LPS_1_-expressing CHO cells and identified LysoPS, but not other LPLs, as a functional ligand for LPS_1_ [42]. They showed that LysoPS mediated a Gαi-dependent inhibition of cAMP production in LPS_1_-expressing CHO cells with an EC_50_ of 270 nM. Similar concentrations of LysoPS also sufficiently induced mast cell degranulation in a Gαi-dependent manner. Based on the strong expression of LPS_1_ in mast cells from rats, mouse, and humans, the authors proposed that LPS_1_ was the LysoPS receptor responsible for mast cell degranulation. However, LysoPS-induced degranulation in mast cells from LPS_1_-deficient mice was similar to that in wild-type mice [43], indicating the involvement of other LysoPS receptor(s). By a chemical approach, Iwashita et al. identified lysophosphatidylthreonine (LysoPT) as a super stimulator of mast cell degranulation [44]. LysoPT resembles LysoPS in structure except for having an additional methyl group but it did not activate LPS_1_, providing further evidence that LPS_1_ is not the LysoPS receptor on mast cells. Liebscher et al. reported that LysoPS did not activate either human or mouse LPS_1_ expressed in yeast and COS-7 cells [43]. Interestingly, however, they showed that LPS_1_ from a fish (carp) nicely responded to LysoPS. Based on these data, the authors argued that LysoPS is not a ligand for human and mouse LPS_1_. Although it is still controversial whether LysoPS is a bona fide endogenous ligand for LPS_1_, Kitamura et al. clearly demonstrated that LysoPS activated LPS_1_ in mammalian LPS_1_-expressing CHO or HEK293 cells using independent three experimental systems: a Gαi-dependent cellular migration assay, a Ca^2+^ response assay, and a TGFα-shedding assay [22]. Of note, TGFα shedding assays showed that LPS_1_ was activated by LysoPS with an unsaturated fatty acid at the *sn*-2 position of the glycerol backbone (*sn*-2 LysoPS) [22] and, as mentioned above, by recombinant PS-PLA_1_, a postulated LysoPS (*sn*-2 LysoPS)-producing enzyme. Thus, LPS_1_ appears to specifically recognize the structure of LysoPS, which clearly shows that it is a cellular receptor for *sn*-2 LysoPS.

In the past decade, several studies have revealed the physiological and pathological functions of LPS_1_ in vivo. Above mentioned Liebscher et al. also showed that LPS_1_ deficiency had immunomodulatory effects in mice [43]. LPS_1_-deficient mice showed exaggerated allergic responses as indicated by increased footpad swelling in delayed-type hypersensitivity (DTH) models. Consistently, splenocytes isolated from LPS_1_-deficient DTH-treated mice exhibited dysregulated production of various pro-inflammatory cytokines, including IL-2, that are known to promote allergic responses, allergic responses under both basal and stimulated conditions. In the mouse model of *Cryptococcus neoformans* infection, LPS_1_-deficiency increased the fungal burden in the lung, brain, and spleen. The same group also reported reduced phagocytosis activity in microglia derived from LPS_1_-deficient mice [45]. More recently, Sayo et al. revealed that LPS_1_ had pathogenic roles in neuropathic pain model mice [46]. In the mice, LPS_1_ was specifically expressed in microglia that reside in the dorsal horn of the spinal cord and was upregulated following nerve injury. The microglia of LPS_1_-deficient mice were comparable in number and morphology to those of wild-type mice but were not activated as judged by their lower production of pro-inflammatory cytokines. This resulted in attenuated neuropathic pain in LPS_1_-deficient mice. The authors also demonstrated that inhibiting LPS_1_ by a specific antagonist protects mice from neuropathic pain. Although the molecular mechanisms underlying these phenotypes in LPS_1_-deficient mice remain unclear, accumulated findings raise the possibility that LPS_1_ has immunomodulatory functions in vivo, possibly by affecting the cellular functions of macrophages, monocytes, and DCs which abundantly express LPS_1_.

Several groups have implicated the role of LPS_1_ in cancer. Several types of cancer, including colorectal cancer [47], cervical cancer [48], marginal zone lymphomas [49, 50], and Bcr-Abl-transformed leukemia cells [51], highly express LPS_1_. In a cervical cancer cell line, downregulation of microRNA-381, which targets 3′-UTR of LPS_1_ to suppress its expression, enhanced the expression of LPS_1_ [48]. Overexpression of LPS_1_ in a gastric cancer cell line enhanced cellular invasion via PI3K/AKT pathways [52]. Similarly, LysoPS treatment stimulated the proliferation and chemotactic migration of colorectal cancer cells, and importantly, those effects were completely abolished by genetic knockdown of LPS_1_. These findings raise the possibility that there is a LysoPS-LPS_1_ axis in cancer cell biology.

## P2Y10/LPS_2_ and A630033H20Rik/LPS_2L_

LPS_2_ was first proposed as an LPA and S1P receptor. Fujita et al. demonstrated that LPA and S1P evoked Ca^2+^ signaling in CHO cells expressing an LPS_2_/Gαl6 fusion protein [53]. However, a subsequent study using TGFα-shedding and actin stress fiber formation assays failed to show the agonistic activity of LPA and S1P against LPS_2_, even under the condition of Gα16 overexpression [54]. Meanwhile, Inoue et al. demonstrated that LysoPS was able to activate LPS_2_. They also showed that structural analogs of LysoPS, which have threonine or D-serine as a polar head, lost the ability to activate the LPS_2_, demonstrating that LPS_2_ strictly recognized the structure of LysoPS. A630033H20Rik/LPS_2L_, the closest homolog of LPS_2_ with 75% sequential homology, also specifically responded to LysoPS, but with slightly less affinity compared to LPS_2_. Since LPS_2L_ is a pseudogene in humans but genetically intact in rodents, LPS_2_ and LPS_2L_ should redundantly function in rodents. While LPS_1_ is functionally coupled to Gαi, LPS_2_ (and also LPS_2L_) shows a strong preference to Gα12/13 downstream signaling [55], suggesting that LPS_2_ has different roles in the cellular responses against LysoPS. Thus, LPS_2_ appears to be a receptor for LysoPS but not for LPA and S1P.

According to the BioGPS database (http://biogps.org), LPS_2_ and LPS_1_ are selectively expressed in immune-related organs, such as the spleen, bone marrow, and lymphoid tissues. Among the immune cells, B cells, T cells, and DCs strongly express LPS_2_ [4, 56]. Rao et al. revealed that the transcription of LPS_2_ is directly regulated by Ets transcription factors PU.1 and Spi-B, which transactivate the various B-cell-related genes during B cell development [56]. Consistently, LPS_2_ is strongly expressed in immature and mature B cells, but not in B cell precursors. This finding suggests the roles of LPS_2_ in certain stages of B cell development. Several immune-related functions of LPS_2_ in these cell types have been revealed in recent years. Kita et al. demonstrated that LysoPS and LPS_2_-selective LysoPS analog suppress LPS-induced TNF-α production in murine DCs and microglia in vitro [7]. Hwang et al. reported LPS_2_ functions in human eosinophils [5]. In in vitro-differentiated eosinophils, a high concentration of LysoPS induces eosinophil degranulation through the activation of ERK, but it does not affect the chemotaxis, differentiation, or survival of eosinophils. The same group also demonstrated that LysoPS induces the formation of an eosinophil extracellular trap (EET) in isolated human eosinophils that strongly express LPS_2_ [8]. The trap consists of eosinophil-derived DNA and functions as a scaffold for pathogenic or protective immune responses. These studies have implicated the potential roles of LPS_2_ in immune cells. However, using tools such as an LPS_2_-specific agonist (see below) and knockout mice will be essential for future LPS_2_ studies.

## GPR174/LPS_3_

LPS_3_ is a Gαs-coupled GPCR that is exclusively expressed in immune-related tissues and cell types, especially in T and B cells [4]. Among LysoPS receptors, LPS_3_ has been the best-characterized in the past few years, and accumulated clinical evidence suggests that LPS_3_ has a role in female-preponderant autoimmune diseases, such as Grave’s disease [57], Addison’s disease [58], and autoimmune thyroid disease [59]. In a genome-wide association study of the X chromosome in a Chinese population, Chu et al. identified an X-linked gene LPS_3_ variant with a non-synonymous single nucleotide polymorphism (SNP), referred to as rs3827440, as a risk factor for Grave’s disease [60]. Similarly, Napier et al. demonstrated a significant association of LPS_3_ variant harboring rs3827440 with Addison’s disease in a UK cohort, indicating that LPS_3_ has immune-related functions [58]. Other studies have also demonstrated the immune-modulatory roles of LPS_3_ in immune cells. Shinjo et al. reported that a LysoPS-LPS_3_ axis suppressed T cell functions via the Gαs pathway [4]. They suggested that LysoPS inhibited the TCR-dependent activation of primary CD4 T cells, as judged by IL-2 production. The inhibitory effect of LysoPS can be completely abolished in LPS_3_-deficient T cells, but not in LPS_1_- or LPS_2_-deficient T cells. Notably, various species of LysoPS can be produced upon activation stimuli. This suggests that a LysoPS-LPS_3_ axis is a cell-autonomous, and intrinsic regulatory mechanism in T cells. A subsequent report by Barnes and Cyster et al. nicely reproduced and strengthened the suppressive roles of LPS_3_ during T cell activation via the Gαs pathway, as indicated by IL-2 production, cell proliferation, and cell-surface expression of T cell activation markers, even in vivo [6].

While these findings demonstrate that LPS_3_ has immune-suppressive roles, LPS_3_ also desuppresses immune responses by inhibiting the function of regulatory T (Treg) cells, which generally act as an immunosuppressor during immune responses [3]. Using the LPS_3_ reporter mice that harbor LPS_3_-coding-exon replaced with dTomato, Barnes et al. found the highest expression of LPS_3_ in Treg cells among T and B cell subpopulation in vivo. Using LPS_3_ reporter mice in which an LPS_3_-coding exon is replaced with dTomato, Barnes et al. [3]. found that the highest expression of LPS_3_ in the T and B cell subpopulations in vivo was in Treg cells. They showed that LPS_3_ negatively regulates the development, proliferation, and functions of Treg cells, thus resulting in the desuppression of effector T cells. Consistently, LPS_3_-deficient mice exhibit an attenuated severity of the experimental autoimmune encephalomyelitis (EAE) model, mainly due to the augmented functions of Treg cells. Similarly, attenuated severities were observed in LPS_3_-deficient mice that had been subjected to LPS-induced sepsis [11]. These findings clearly demonstrated that LPS_3_ had cell type-dependent immunomodulatory functions.

More recently, Ruozhu et al. revealed that the function of LPS_3_ in B cells during the formation of germinal center (GC) was sex-specific [9]. GC is a specific region in the lymph node where antigen-activated B cells undergo differentiation into the antibody-secreting plasma cells during the immune responses against a foreign antigen. LPS_3_ inhibits the localization of B cells to the follicle center, in which B cells aggregate to form the GC, thus preventing GC formation. Interestingly, this phenotype was observed only in males, possibly because the B cells of females have a higher capacity to form GCs [11]. In the same study [11], the authors identified a chemokine (CCL21), but not LysoPS, as an endogenous ligand of LPS_3_. Notably, CCL21 activates Gαi-signaling to regulate the migration of B cells, whereas LysoPS activates Gαs-signaling to regulate the functions of T cells, which suggests that there is a cell type- and ligand-dependent bias in downstream Gα protein signals.

## Other LysoPS Receptors

In addition to the four P2Y-type GPCRs, G2A also referred to as GPR132, is a possible receptor for LysoPS [61, 62]. Like other LysoPS receptors, G2A is highly expressed in B cells, T cells, and macrophages and is upregulated in response to cellular stress stimuli such as DNA damage [63]. It was initially identified as a receptor for lysophosphatidylcholine (LPC). Later studies demonstrated that G2A can respond to extracellular protons [64], 9-hydroxyoctadecadienoic acid (9-HODE, a species of oxidized free fatty acid) [65], and LysoPS [62]. In human neutrophils, LysoPS and lysophosphatidylethanolamine (LPE) evoke a calcium response [66]. During neutrophil apoptosis, LysoPS is produced in an NADPH-mediated manner and enhances the engulfment of apoptotic neutrophils by macrophages. These effects of LysoPS are canceled by treatment with an anti-G2A antibody, which suggests that G2A is involved in LysoPS signaling. However, in the absence of information on the structure and the structure-activity relationship (SAR) of G2A, it is unclear whether G2A is a genuine, direct LysoPS receptor. It should be noted that G2A was once proposed as a cellular receptor for LPC, but the report was retracted. Thus, a more precise characterization of the receptor is needed in the future.

In schistosome-infected DCs, toll-like receptor 2 (TLR2) was activated by schistosome-derived LysoPS, which identified TLR2 as a non-GPCR-type LysoPS receptor [39]. TLR2 was also identified as a non-GPCR-type LysoPS receptor in primary peritoneal macrophages treated with 1 μM LysoPS [15]. Although they did not show the direct binding of LysoPS to TLR2, TLR2-deficient macrophages showed significantly reduced response to LysoPS stimulation [15], suggesting that TLR2 is a cellular receptor for LysoPS and that LysoPS-TLR2 signaling is involved in the induction of proinflammatory responses.

## LysoPS Receptor Modulators (Agonists and Antagonists)

Since many of the biological actions of LysoPS are exerted through GPCRs, as mentioned above, GPCR modulators (selective agonists and antagonists) could be attractive tools for the elucidation of LysoPS biology. Although LPS_1_ agonists and antagonists have been identified by screening a chemical library (Patent US 2010/0130737A1, 27 May 2010) [67], no small-molecule modulators are presently commercially available for any LysoPS receptors. The chemical synthesis of structural analogs of LPLs is expected to be an alternative strategy for developing receptor modulators and has been especially successful for LPA and S1P [68, 69]. Thus, in the past decade, our group has been developing a series of LysoPS-like compounds (LysoPS analogs), in which the *sn*-1 or *sn*-2 hydroxy, acyl chain, glycerol, and serine group were chemically modified (Table 2). SAR analyses using these compounds have provided valuable clues for optimizing chemical modules of LysoPS analogs to improve their selectivity and potency against each LysoPS receptor. For example, modification of an *sn*-2 hydroxyl group dramatically reduced its agonistic activity, especially against LPS_3_ [70]. The fatty acid chain length and the number of double bonds in LysoPS analogs influenced their abilities to activate LysoPS receptors but to different degrees for each receptor. Modification of the ester linkage on the glycerol backbone and the serine moiety also affected receptor selectivity. By combining the chemical modules obtained from the SAR analyses, Ikubo et al. were able to create potent agonists that selectively activated LPS_2_ or LPS_3_ at nM concentrations [71]. Moreover, computational virtual docking based on a SAR study predicted the mechanism by which LysoPS analogs were recognized by LPS_1_ on the cell membrane, which will help the further development of more potent LPS_1_ agonists [72–74].

Subsequent researches have introduced more intensive modular structures. The conformation of three carbon atoms of the glycerol moiety of LysoPS and conventional LysoPS analogs have three-dimensional flexibility. To limit the conformational flexibility, Jung et al. designed and synthesized a series of LysoPS analogs in which various ring structures were introduced into the glycerol backbone [75]. Intriguingly, not only the type of chemical group attached to the ring structure but also its orientation in three dimensions significantly influenced the agonistic activity against LPS_1_ and LPS_3_. In 2009, LysoPT and its deoxy derivative of the *sn*-2-hydroxyl group were found to strongly promote antigen-elicited mast cell degranulation while LysoPS did not [44]. These LysoPS analogs could evoke mast cell-dependent hypothermia in mice. Later, by modifying the fatty acid moiety of LysoPT, a more potent compound (2-deoxy-1-C3-pH-p-O-C11-LysoPT) with mast cell degranulation activity at nM concentration was synthesized [76]. Notably, all these LysoPS analogs lacked agonistic activity against conventional LysoPS receptors, indicating the presence of an uncharacterized recognition mechanism for LysoPS in mast cells. Collectively, these LysoPS analogs would help to unravel the biology behind LysoPS receptors both in vivo and in vitro and potentially be useful in treating immunological diseases.

## Clinical Implications

**Fig. 2:**
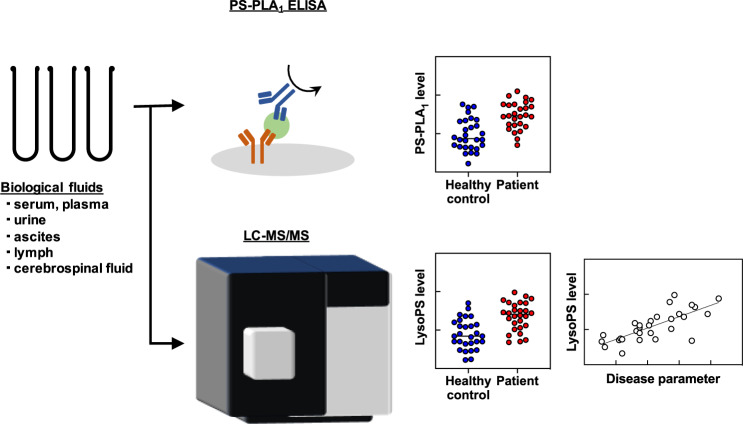
Possible clinical application of LysoPS and PS-PLA_1_ as a biomarker. PS-PLA_1_ is the secreted enzyme and thus can be measured by ELISA in the various biological fluids. LysoPS can also be quantified using the highly sensitive LC-MS/MS system. Recent clinical studies revealed the elevated levels of LysoPS and PS-PLA_1_ in the various clinical samples, suggesting the potential utility of these molecules as a potential biomarker

The recent development of techniques for analyzing clinical samples has led to many insights into the pathophysiological roles of LysoPS [77–82]. A possible LysoPS-producing enzyme, PS-PLA_1_, is secreted extracellularly and is detected in various biological fluids, including serum. Yatomi et al. established a PS-PLA_1_ immunoassay [83] and subsequently reported that PS-PLA_1_ antigen concentrations were variable in clinical samples from patients (Fig. 2). In healthy individuals, serum PS-PLA_1_ concentration was about 30 µg/L and was slightly higher in men than in women [83]. Serum PS-PLA_1_ was significantly elevated in patients with autoimmune disorders, including systemic lupus erythematosus (SLE), rheumatoid arthritis, and sjögren’s syndrome [82]. Interestingly, the level of PS-PLA_1_ in each SLE individual showed a good association with the SLE disease activity index and decreased after the start of medical therapy. Similar results were observed in hyperthyroidism, in which PS-PLA_1_ levels varied with disease onset and treatment [81]. Therefore, it is assumed that PS-PLA_1_ and possibly its product LysoPS contribute to the development and progression of these immune disorders.

The current development of LC-MS makes it possible to detect and quantify less abundant LPLs, including LysoPS, in biological samples. Unlike S1P, another bioactive LPL present in the circulation at sub-micromolar concentrations, under healthy conditions [84, 85], plasma LysoPS reported so far was at a low level (approximately several 10 nM) [79]. The concentration of LysoPS was suboptimal for LysoPS receptor activation, suggesting that LysoPS is produced locally and that its production depends on pathological conditions [54]. Activated platelets might be another source of LysoPS [86]. In fact, the level of LysoPS in platelet-rich plasma was much higher than that in platelet-poor plasma [87]. Plasma LPLs, including LysoPS, a mostly unsaturated form of LPL, was elevated in patients with the acute coronary syndrome (ACS) that is caused by platelet activation followed by atherosclerotic responses [80]. Intriguingly, among various LPLs, only LysoPS was positively correlated with serotonin, a clinical marker of platelet activation [79]. Activated platelets expose PS on the outer membrane. In addition, PS-PLA_1_ is abundantly expressed in platelets at least in rodents. These notions suggest that PS-PLA_1_ is responsible for the production of LysoPS from platelets. Kurano et al. also suggested the possibility that LysoPS is produced in a PS-PLA_1_-independent manner in ACS [80]. Thus, plasma LysoPS might be a reliable marker of platelet activation, although its pathological role in atherosclerotic diseases is unclear. LysoPS was detected in the tumors and cancerous ascites [78]. In colorectal cancers, the total level of LysoPS species was significantly increased, whereas that of LPA was decreased [88]. Similarly, LysoPS levels in ascites were markedly higher in gastric cancer patients [78]. Various cancer cells express PS-PLA_1_ as well as LPS_1_, and their expression is particularly elevated in hepatocellular carcinoma, suggesting that a PS-PLA_1_-LysoPS-LPS_1_ signaling axis is involved in tumorigenesis [77].

## Conclusions and Future Prospects

Despite its early discovery in the 1950s, LysoPS studies have lagged behind those of the two major LPLs, S1P and LPA, leaving many questions unanswered about its tissue and cellular distribution, producing enzymes, and receptors. In the past decade, we have had the answers to these questions, and now LysoPS is being recognized as an essential bioactive lipid. In addition, we now have various research tools for LysoPS signaling including LC-MS system to detect LysoPS, ELISA system to quantify LysoPS-producing enzymes, knockout animals, and agonists and antagonists to modulate specific receptor functions. Researches to date have implicated the possible roles of LysoPS in immune-related pathophysiology. The next challenge in this field is to reveal the precise mechanism of action of the LysoPS-LysoPS receptor axis in various immune-related pathophysiological conditions such as cancer, autoimmune diseases, and infectious diseases. A better understanding of the mechanisms, especially in vivo, should help to develop therapeutics against these diseases.
